# Ligand‐specific recycling profiles determine distinct potential for chronic analgesic tolerance of delta‐opioid receptor (DOPr) agonists

**DOI:** 10.1111/jcmm.15234

**Published:** 2020-04-12

**Authors:** Hanieh Bagheri Tudashki, Youssef Haddad, Iness Charfi, Rejean Couture, Graciela Pineyro

**Affiliations:** ^1^ Centre de Recherche Centre Hospitalier Universitaire Ste-Justine Montréal QC Canada; ^2^ Department of Pharmacology and Physiology Faculty of Medicine Université de Montréal Montréal QC Canada

**Keywords:** analgesic response, opioid, tolerance

## Abstract

δ‐opioid receptor (DOPr) agonists have analgesic efficacy in chronic pain models but development of tolerance limits their use for long‐term pain management. Although agonist potential for inducing acute analgesic tolerance has been associated with distinct patterns of DOPr internalization, the association between trafficking and chronic tolerance remains ill‐defined. In a rat model of streptozotocin (STZ)‐induced diabetic neuropathy, deltorphin II and TIPP produced sustained analgesia  following daily (intrathecal) i.t. injections over six days, whereas similar treatment with SNC‐80 or SB235863 led to progressive tolerance and loss of the analgesic response. Trafficking assays in murine neuron cultures showed no association between the magnitude of ligand‐induced sequestration and development of chronic tolerance. Instead, ligands that supported DOPr recycling were also the ones producing sustained analgesia over 6‐day treatment. Moreover, endosomal endothelin‐converting enzyme 2 (ECE2) blocker 663444 prevented DOPr recycling by deltorphin II and TIPP and precipitated tolerance by these ligands. In conclusion, agonists, which support DOPr recycling, avoid development of analgesic tolerance over repeated administration.

## 1 INTRODUCTION

δ‐opioid receptors (DOPrs) have emerged as attractive targets for the treatment of chronic pain syndromes.^1^ They display analgesic efficacy in pre‐clinical models of inflammatory^2^ and neuropathic pain,^3^, ^4^, ^5^ whereas their anxiolytic‐ and antidepressant‐like actions^6^ provide a means of managing emotional distress associated with these conditions.^7^ In addition, DOPr agonists have less potential for abuse than µ‐opioid receptor (MOPr) agonists^8^, ^9^ and their side‐effects profile is milder, particularly in terms of respiratory depression,^8^, ^10^ constipation^8^, ^11^ and physical dependence.^12^, ^13^ However, despite these advantages repeated administration of DOPr agonists may induce analgesic tolerance,^14^, ^15^ limiting their effectiveness for long‐term management of chronic pain.

Interestingly, the potential for inducing tolerance differs across DOPr agonists,^16^, ^17^, ^18^ suggesting that this undesired effect could be mitigated through rational drug design. Given this possibility, considerable effort has focused in understanding cellular and molecular mechanisms underlying the decline of analgesic responses to DOPr ligands, especially how ligand‐induced trafficking contributes to development of tolerance.^14^, ^18^ In particular, single exposure to ‘highly internalizing’ agonists like SNC‐80 but not ‘low‐internalizing’ agonists like ARM390 was shown to abolish analgesic response to a single subsequent injection of the corresponding agonist.^18^ These observations led to the proposal that ligand‐specific sequestration patterns could be predictive of ligand potential to induce acute analgesic tolerance.

However, to interpret functional consequences of receptor internalization we must also consider the post‐endocytic fate of internalized receptors. For example, a simple association between acute tolerance and sequestration efficacy does not hold true for deltorphin II^17^ or DPDPE, both of which produce DOPr internalization comparable to SNC‐80,^16^, ^19^ but do not produce acute tolerance. We have previously shown that the potential of DPDPE to induce acute tolerance is minimized by its capacity to support receptor recycling to the membrane, which does not occur with SNC‐80.^16^, ^19^ Internalization patterns per se have also failed to account for development of chronic tolerance as low and high internalizing ligands similarly result in loss of analgesic response over repeated administration.^14^, ^15^ On the other hand, the extent to which receptor recycling influences development of chronic analgesic tolerance to DOPr agonists remains to be determined.

In the present study, we sought to answer this question and found that DOPr agonists that supported receptor recycling to the membrane (deltorphin II; TIPP) induce sustained analgesia, independent of their efficacy or patterns of low (TIPP) or high (deltorphin II) internalization capacity. In contrast, ligands like SB235863 and SNC‐80, which did not support membrane recovery of internalized receptors, displayed progressive loss of analgesic actions irrespective of the degree of internalization they induced or of the analgesic efficacy they displayed. The association between sustained analgesia and recycling was further demonstrated by the fact that inhibition of DOPr recycling precipitated tolerance in TIPP‐ and deltorphin II‐treated rats.

## 2 MATERIALS AND METHODS

### 2.1 Materials

Drugs were purchased from different companies: deltorphin II was from AnaSpec, SNC‐80 ((+)‐4‐[(*αR*)‐*α*‐((2*S*,5*R*)‐4‐allyl‐2,5‐dimethyl‐1‐piperazinyl)‐3‐methoxybenzyl]‐*N*,*N*‐diethylbenzamide) was from Tocris Cookson, SB235863 ([8*R*‐(4bS*,8aα,8a*β*,12b*β*)]7,10‐dimethyl‐1‐methoxy‐11‐(2‐ethylpropyl)oxycarbonyl 5,6,7,8,12,12b‐hexahydro‐(9*H*)‐4,8‐methanobenzofuro[3,2‐e]pyrrolo[2,3‐g]isoquinoline hydrochloride) was a generous gift from Dr L Gendron^17^ (University of Sherbrooke, QC, Canada), and TIPP was from Cedarlane. Streptozotocin (STZ) was purchased from Cayman Chemical. The endothelin‐converting enzyme 2 (ECE2) inhibitor, 6634449, was purchased from Vitas‐M laboratory (product code STK521587).

### 2.2 Animals

Adult male Sprague Dawley (SD) rats, weighing 235‐250 g, were purchased from Charles River laboratories and housed in a controlled environment on a 12‐hour light/dark cycle with free access to food and water. All experimental methods and animal care procedures were approved by the Animal Care Committee of University of Montreal (CDEA protocol 15‐013), in accordance with the guiding principles as enunciated by the Canadian Council on Animal Care.

### 2.3 Induction of diabetic neuropathic pain

Type I diabetes was induced by systemic injection of streptozotocin (STZ, 65 mg/kg, i.p.) according to a standardized methodology, which allows development of persistent diabetic neuropathy after the first week of treatment.^20^ At the end of the first week, blood was extracted from the tail vein and blood glucose levels were measured with a glucometer (Accu‐Chek Aviva; Roche Diagnostics) prior to evaluation of analgesia on the second week. Diabetes was confirmed when blood glucose concentration was between 20 and 28 mmol/L.^21^, ^22^ STZ‐induced diabetes displays clear sensory abnormalities mimicking human neuropathy such as mechanical and cold allodynia,^22^ which were previously shown to respond to DOPr agonists.^23^

### 2.4 Assessment of mechanical allodynia

Mechanical allodynia is the consequence of maladaptive neuroplasticity following nerve damage.^24^ In a model of diabetic neuropathy as the one used in this study, hyperglycaemia contributes to generate reactive oxygen species which induce microglia activation and inflammatory mediators.^25^, ^26^ These mediators attenuate the activity of Gly/GABA interneurons in superficial layers^24^, ^27^, ^28^, ^29^ and disrupt the inhibitory control that prevents innocuous touch stimuli from producing pain which leads to allodynia.^30^ DOPrs are expressed in these light‐touch mechanoreceptors^31^ and up‐regulated during sensitization,^32^, ^33^ making DOPr agonists elective for relieving this type of pain. Allodynia was evaluated using Von Frey filaments as previously described.^19^ Briefly, rats were accustomed for 15 min on a metal mesh floor under an inverted plastic box (20 × 10 × 10 cm) in a quiet room dedicated to this behavioural test. After habituation, the plantar surface of the right and left hind legs was alternately touched (6‐8 seconds) with von Frey filaments of progressively wider diameter in order to find the threshold of pressure required to produce withdrawal. The mechanical threshold response was obtained by consigning the pressure in grams that would result in withdrawal of the paw in 50% of 10 trials. A normal rat responds to tactile pressures of 12‐15 g whereas neuropathic rats respond to pressures of 4‐6 g.^24^

### 2.5 Pharmacological treatments

In a first series of experiments, dose‐response curves to SNC‐80, SB235863, deltorphin II and TIPP were constructed following intrathecal (i.t.) injection of increasing doses until no increment in maximal anti‐allodynic response was observed. Deltorphin II and TIPP are peptidic ligands^17^, ^34^, ^35^ with poor blood‐brain barrier penetration such that i.t. administration ensured that all ligands reached the desired targets within spinal cord and dorsal root ganglia in primary afferents. Each group of rats received only one ligand and a single dose per day. Saturation of the analgesic response on mechanical allodynia required assessing 5‐6 doses per ligand administered  over a period of 5‐6 days, using 3‐4 animals/dose. Injections were made at mid‐lumbar level with a needle 23G × 1/2 under light anaesthesia using 4% isoflurane inhalation in a 1:1 mixture of oxygen and air. The animals were allowed to recover for 15 minutes before mechanical thresholds were determined. Threshold measures were repeated every 15 minutes until the analgesic response disappeared. Peak analgesic responses (30 minutes) or responses integrated over time (area under the curve: AUC) were consigned and plotted as a function of treatment dose. Curves were fitted with the four‐parameter logistic equation in GraphPad 7 to yield ED_80_ and ED_50_ values that were then used to treat rats for assessment of chronic tolerance. For each drug, the stock solution was prepared as follows: deltorphin II was dissolved in sterile artificial cerebrospinal fluid (CSF); TIPP and SB235863 stocks were dissolved in sterile water and SNC‐80 in DMSO. Each stock solution was diluted in CSF at required concentrations and injected i.t. in a final volume of 30 μL. Control animals were injected with 30 μL of CSF. At the end of behavioural experiments, rats were anaesthetized by isoflurane and killed by decapitation.

### 2.6 Assessment of chronic tolerance

**Figure 1:**
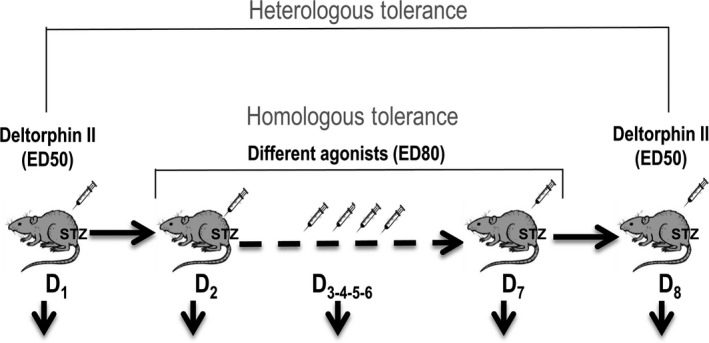
Experimental design. One week after STZ administration, repeated treatments with DOPr agonists were started. Before starting chronic treatment with ED_80_ of deltorphin II, TIPP, SB235863 or SNC‐80, animals were tested to establish the baseline allodynia. Immediately after, they were injected with deltorphin II at a dose corresponding to ED_50_, which was repeated at the end of treatment. Allodynia measures were taken every 15 min after each injection until analgesic effects disappeared. Chronic tolerance was evaluated by comparing analgesic response to successive i.t. injections of each agonist between days 2 and 6. Difference in analgesic response to deltorphin II ED_50_ in days 1 and 8 allowed to establish tolerance induced by the different agonists to this common probe

To evaluate homologous tolerance, each agonist was injected i.t. once daily for six consecutive days, at ED_80_ derived from dose‐response curves (deltorphin‐II_ED80_: 0.30 nmol/30 μL; TIPP_ED80_: 0.40 nmol/30 μL; SB235863_ED80_: 1.7 nmol/30 μL and SNC‐80_ED80_: 0.55 nmol/30 μL). Immediately after drug administration, mechanical allodynia was measured every 15 minutes until the analgesic response disappeared. Tolerance to a common probe was also evaluated, by comparing analgesic effects of a challenge dose of deltorphin II (ED_50_: 0.23 nmol/30 μL), which was given the day prior and the day after 6‐day treatment with the different agonists (diagram of experimental design in Figure 1). Prior to starting any pharmacological treatment, the presence of allodynia was confirmed. Treatments and measures were carried out randomly by two different experimenters to ensure blinding to treatment for the person assessing allodynia. In a series of experiments, an inhibitor of endothelin‐converting enzyme 2 (ECE2), 6634449, was administered by i.t injection (3 nmol/30 μL) 20 minutes prior to administration of DOPr ligands from 2 to 7 days. The vehicle (4% DMSO diluted in CSF sterile) was given as control. The dose of 6634449 used in this study was based on results previously published.^25^

### 2.7 Primary neuronal cultures

Primary neuronal cultures were prepared from rat post‐natal prefrontal cortex (P0‐P2) as previously described.^19^ Briefly, three cultures were independently prepared from 8 to 12 pups each. Pups were cryo‐anaesthetized, and brains were removed and transferred into ice‐cold dissociation solution (NaS0_4_ 90 mmol/L; K_2_SO_4_ 30 mmol/L; MgCl_2_ 5.8 mmol/L; CaCl_2_0.25 mmol/L; HEPES 10 mmol/L; glucose 20 mmol/L; pH 7.4). Following dissection, the frontal cortex was digested in papain solution (20 U/mL; 40 minutes at 37°C), and the product passed through Pasteur pipettes of progressively decreasing diameter for mechanical dissociation. The suspension obtained was centrifuged, and cells were then resuspended and diluted to a density of 2.5 million/mL, before plating onto glass coverslips pre‐coated with collagen/poly l‐lysine (each at 0.1 mg/mL). Culture proceeded in supplemented Neurocell medium (B27 4%; 100 U/mL penicillin‐streptomycin; GlutaMAX 2%; FBS 10%) for 24 hours. Coverslips were then transferred to a six‐well plate containing 2 mL of Neurocell medium/well and transfected with Flag‐DOPr (8 µg/well), using a calcium phosphate transfection protocol previously.^19^ Murine DORs tagged with the Flag epitope at the N terminus were a generous gift from Dr M. von Zastrow (University of California at San Francisco, San Francisco, CA).

### 2.8 Labelling and quantification of DOPr trafficking in primary neuron cultures

Immunolabelling of surface receptors for quantification of trafficking was performed as previously described.^19^ Briefly, primary cultures expressing Flag‐DOPrs were incubated at 37°C with Neurocell medium containing Ca^2+^‐dependent mouse anti‐Flag M1 antibody (1:100; Sigma). After 30 minutes incubation with the antibody, vehicle (0.05% DMSO in Neurocell) or agonist (TIPP, deltorphin II; SNC‐80; SB235863; 10 μmol/L) was added to the medium for 60 minutes. At the end of this period, cultured neurons were washed at 37°C once in calcium‐free PBS and then in PBS in order to remove treatment agonist as well as antibody bound to receptors that may have remained at the membrane after agonist exposure. For visualization of internalized receptors, cultures were immediately fixed with 4% PFA, permeabilized with PBS/0.1% Triton (20 minutes at room temperature (RT)), blocked with PBS/BSA 1% (10 minutes at RT) and incubated with secondary antimouse Alexa 488‐conjugated donkey antibody (1:1000; Invitrogen, A21202). Alternatively, cultured neurons were allowed to recover for 60 minutes in the absence of ligand before a second round of calcium‐free PBS wash was completed before fixation, permeabilization and incubation with secondary antibody. The latter procedure allowed to remove antibody bound to Flag‐DOPrs that translocated to the surface during recovery ensuring the exclusive labelling of receptors that were retained intracellularly.^26^ Another set of neurons was similarly treated with agonist or vehicle and then allowed to recover for 60 minutes following which they were fixed and incubated with secondary antibody without permeabilization, so as to exclusively reveal Flag‐DOPrs that reappeared at the surface during recovery from internalization; measures of surface labelling were normalized to intracellular labelling produced during internalization. Recycling was thus established by taking two independent measures: (a) cytoplasmic labelling density lost during recovery from treatment and (b) gain in surface labelling density when neurons were allowed to recover from agonist‐induced internalization. In experiments in which 6634449 was introduced in the medium during recovery, the concentration used was 20 μmol/L.

Cytoplasmic and surface labelling densities were quantified with ImageJ using a previously described method,^27^ with small modifications.^16^ In particular, total cytoplasmic labelling density was obtained by measuring fluorescence intensity within the region confined between the external and nuclear perimeters, and dividing this value by the corresponding area. Total surface labelling density was defined by calculating the ratio of fluorescence measured within internal and external perimeters of surface‐labelled neurons, and the corresponding area. Nuclear labelling density (fluorescence within nuclear perimeter/nuclear area) was considered background and subtracted from total density values just described. Contours defining each of the regions of interest were first drawn on brightened images, and once the trace was completed, brightness was reset to acquisition conditions. Images were acquired with a FluoView 1000 confocal laser‐scanning microscope (Olympus) using a 60× objective. Gain was set for each independent experiment, using calibration slides. These consisted of vehicle‐ or SNC‐80‐treated cultures processed for intracellular labelling. Calibration was performed by adjusting gain to minimize saturation in the internalization slide while still being able to visualize intracellular labelling in the vehicle slide. Once the parameters were set, they were kept constant across all conditions in the same experiment to ensure that differences in labelling density represented differences in receptor density.

### 2.9 Data analysis

Data analysis was performed using GraphPad 7 (GraphPad Software). Statistical comparisons are detailed in the text or in figure legends.

## 3 RESULTS

### 3.1 Acute analgesic responses of ‘low‐internalizing’ agonists last longer than those of ‘high internalizing’ ones

**Figure 2:**
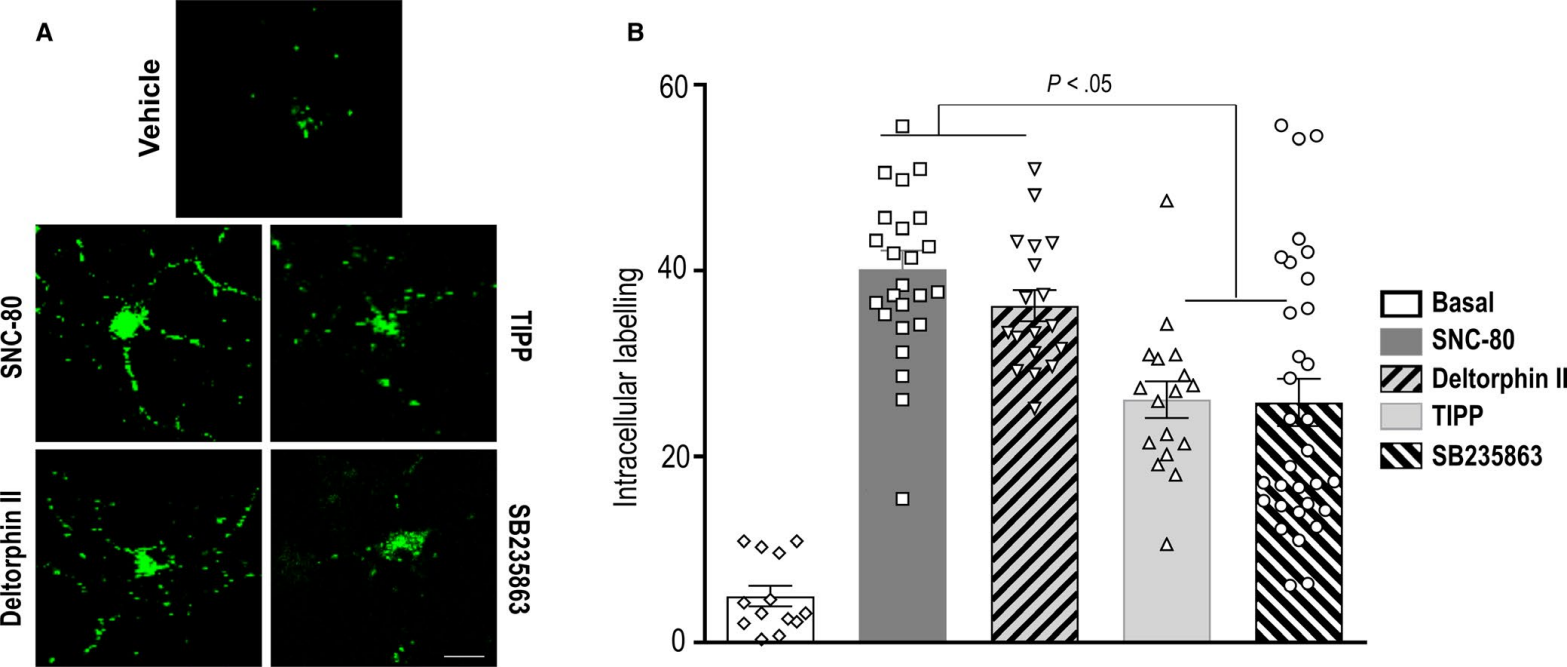
Agonist‐induced internalization in DOPrs expressed in cortical neurons. A, Primary cortical neuron cultures were transfected with Flag‐DOPrs and incubated with primary antibody prior to exposing neurons to vehicle (neurobasal) or to agonists (10 µmol/L, 60 min) as indicated. By the end of treatment, drugs were washed out, antibody bound to receptors remaining at the cell surface was stripped, and cells were immediately processed to reveal receptors that translocated from the cell membrane to the intracellular compartment following constitutive (vehicle) or ligand‐induced sequestration. B, Histograms show intracellular labelling density ± SEM (arbitrary units). Data were generated in three independent experiments, and the total number of neurons quantified per condition is shown in the figures. Statistical significance was established by one‐way ANOVA followed by Sidak's post hoc test. The analysis revealed all ligands induced a significant increase in intracellular labelling as compared to vehicle (*P* < .001). Results of statistical analysis for comparisons among ligands are shown in B

We have previously established that internalization by DOPr agonists could predict the rate of decay of peak signalling in in vitro* assays*.^36^ In particular, when tested in HEK cells agonists like SB235863 and TIPP induced minimal internalization and slow decay of cAMP inhibition. In contrast, SNC‐80 and deltorphin II produced marked internalization and fast decay in cAMP inhibition.^36^ Here, we wanted to determine whether internalization profiles had any predictive value over the duration of acute analgesic responses. As internalization capacity of different agonists differs in HEK cells and neurons,^37^ we first examined whether the relative internalization profiles described for SB235863, TIPP, SNC‐80 and deltorphin II in HEK cells could be retrieved in neurons. To do so, primary cortical cultures were transfected with Flag‐DOPrs, and on the day of the experiment, receptors were labelled at the cell surface followed by exposure to a maximal effective concentration (10 µmol/L; 60 minutes) of the different agonists of interest. Treatment with the different agonists caused DOPrs labelled at the neuron surface to translocate and accumulate in the intracellular compartment. As shown in Figure 2, sequestration induced by maximal effective concentrations of SNC‐80 and deltorphin II was significantly higher than that induced by SB235863 and TIPP, corroborating that the relative internalization profiles described in HEK cells could also be observed in neurons.

**Figure 3:**
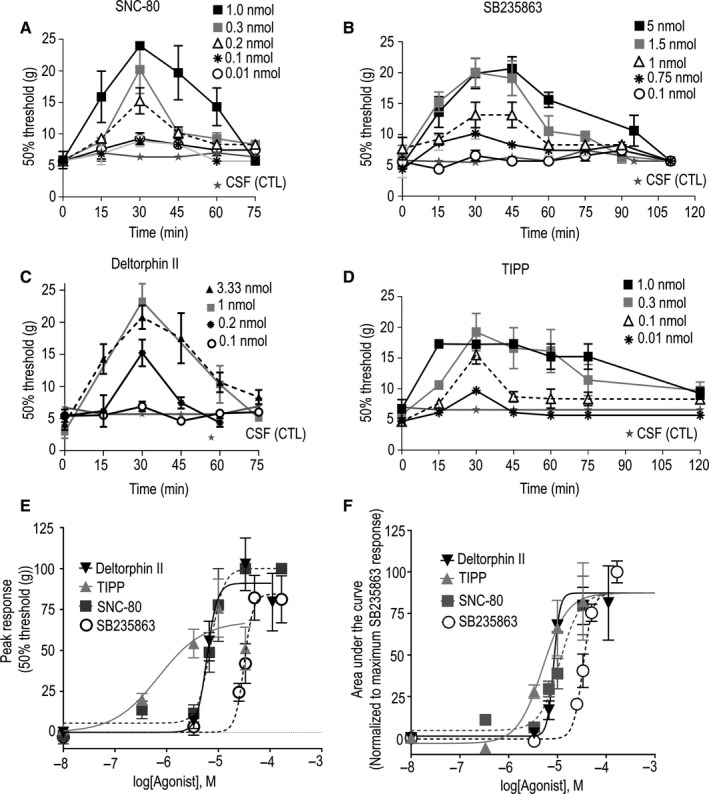
Duration of acute analgesic responses is different for high‐ and low‐internalizing agonists. One week after STZ administration, rats received i.t. injections of vehicle (CSF) or (A) SNC‐80, (B) SB235863, (C) deltorphin II or (D) TIPP at the indicated doses. Mechanical thresholds were assessed immediately after agonist injection and then every 15 min until return to baseline. Results correspond to pressure withdrawal threshold (mean ± SEM) of 3‐4 rats per concentration point. E, Analgesic response observed 30 min after injection is plotted as function of dose. F, The analgesic response induced by different doses was integrated from the time of the first measure until analgesic effect disappeared (area under the curve, AUC). AUC values were plotted as a function of dose. Parameters describing each dose‐response curve were derived with the four‐parameter logistic equation. Curve parameters for different agonists were compared using global curve fitting with shared parameters (GraphPad 7). EC_50_ and Emax values are shown in Table 1 and discussed in the text

To determine the duration of the analgesic response induced by agonists with different internalization capacity, we measured their ability to reduce mechanical allodynia in a model of diabetic neuropathy that had been previously shown to respond to DOPr agonists.^23^ All four ligands produced a dose‐dependent increase in the threshold pressure that was necessary to evoke mechanical allodynia in streptozotocin (STZ)‐treated rats. However, there were differences in duration (Figure 3A‐E) and in maximal responses attained after each treatment (Figure 3F). Thus, if for each ligand we consider analgesia produced by doses that reached the maximal effects the responses elicited by SNC‐80 (Figure 3A; one‐way ANOVA vs control at corresponding time‐point *P* = .0909; n = 3) and deltorphin II (Figure 3C; *P* = .1196; n = 3‐4) were no longer significantly different from vehicle 60 min after injection. In contrast, analgesia by doses which elicited maximal SB235863 (Figure 3B; *P* = .0028; n = 3‐4) and TIPP (Figure 3D; *P* < .0001; n = 3‐4) responses was still present at this time‐point. Such differences in time course implied that the analgesic response integrated over time (area under the curve) was comparable for all four agonists (Figure 3E and Table 1), despite considerable differences in potency and maximal effect (Figure 3F). Indeed, the maximal analgesic response by TIPP was significantly more potent (EC_50_ TIPP vs EC_50_ SNC‐80: *P* = .0048) but its maximal effect was smaller than that of SNC‐80 (E_MAX_ TIPP vs E_MAX_ SNC‐80: *P* = .0017; 23 and 16 degrees of freedom, respectively). In turn, SNC‐80 was more potent than SB235863 (EC_50_ SB235863 vs EC_50_ SNC‐80: *P* = .0072; 18 and 16 degrees of freedom, respectively), but not different from deltorphin II **(**EC_50_ Delt. II vs EC_50_ SNC‐80: *P* = .5210; E_MAX_ Delt II vs E_MAX_ SNC‐80: *P* = .4336; 14 and 16 degrees of freedom, respectively**)** (Figure 3F). Taken together, these observations show that similar to in vitro signalling responses,^36^ the duration of analgesia by DOR agonists was longer for low‐internalizing agonists with low efficacy/potency profiles (TIPP and SB235863) as compared to highly internalizing efficacious ligands (SNC‐80 and deltorphin II).

**Table 1:** Logistic parameters describing anti‐allodynic effect of DOPr agonists^a^

Agonist	Peak analgesic response		Area under the curves	
	Relative^b^ E_MAX_ ± SEM	pEC50 ± SEM (M)	Relative^b^ E_MAX_ ± SEM	pEC50 ± SEM (M)
SNC‐80	100 ± 7.8	5.15 ± 0.06	87.9 ± 22.1	4.95 ± 0.18
Deltorphin II	91.0 ± 9.0	5.22 ± 0.86	80.5 ± 8.0	5.09 ± 0.05
SB235863	84.4 ± 8.8	4.50 ± 0.05	100.6 ± 5.1	4.44 ± 0.03
TIPP	67.7 ± 9.1	6.15 ± 0.40	84.29 ± 17.3	5.33 ± 0.18

^a^ Logistic parameters were calculated from curves shown in Figure 2E,F.

^b^ E_MAX_ values were normalized to maximal asymptote of agonist with largest effect.

### 3.2 Recycling profiles are associated with persistent analgesia over repeated drug administration

**Figure 4:**
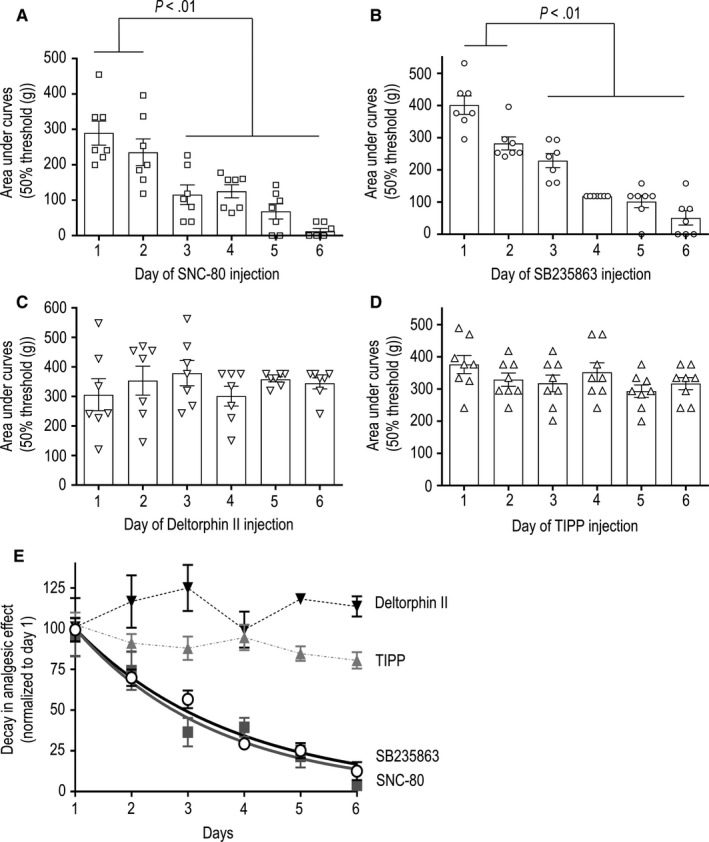
DOPr agonists induce different degrees of chronic tolerance over repeated administration. One week after rats were rendered diabetic by STZ injection, (A) SNC‐80 (0.55 nmol/30 μL), (B) SB235863 (1.7 nmol/30 μL), (C) deltorphin II (0.30 nmol/30 μL) or (D) TIPP (0.40 nmol/30 μL) was administered i.t. at ED_80_ during six consecutive days. Pressure withdrawal thresholds for mechanical allodynia were measured at 15‐min intervals immediately after each administration until return to baseline, and the area under the curve was consigned each day. Results are expressed as mean area ± SEM and correspond to seven rats/group. Statistical comparisons were performed using one‐way ANOVA to reveal a difference in the effect of daily injections of SNC‐80 (*P* ˂ .0001) and SB235863 (*P *˂ .0001), but not for deltorphin II (*P* = .6626) or TIPP (*P* = .2145) treatments. Post hoc comparisons using Sidak's test revealed differences in the effect of successive injections of SNC‐80 and SB235863 as indicated in the figure. E, Graph shows the time course of results shown in A‐D, where analgesia by consecutive injections was normalized to the effect of the first injection of each corresponding ligand. Only the kinetics of SNC‐80 and SB235863 responses could be fit to an exponential decay (details in text)

Because internalization profiles were associated with distinct duration of the analgesic response induced by a single injection of DOPr agonists, we were interested in establishing whether these same profiles would be predictive of the persistence of analgesic responses over repeated injections. To address the question, rats received six consecutive i.t. injections of either SNC‐80, deltorphin II, SB235863 or TIPP administered at 24‐hour intervals, and anti‐allodynic effects of each drug were monitored after each injection (Figure 4A‐D). Repeated administration was carried out at ED_80_ doses for each ligand. As expected from previous studies,^14^, ^18^ the anti‐allodynic effect of SNC‐80 gradually decreased over a 6‐day treatment (Figure 4A). Interestingly, this was not the case for the other highly internalizing agonist deltorphin II (Figure 4C), which maintained its analgesic response over six consecutive days of treatment. Development of analgesic tolerance over repeated administration also differed between the two agonists with low internalization profiles. In particular, the analgesic response to SB235863 was practically abolished over repeated administration (Figure 4B), whereas analgesia by TIPP was maintained throughout treatment (Figure 4D). It is also worth noting that chronic tolerance developed at similar rate for SNC‐80 (*t*_1/2_: CI = 1.05‐2.19 days) and SB235863 (*t*_1/2_: CI = 1.69‐2.23 days; Figure 4E), independent of their distinct internalization profiles.

**Figure 5:**
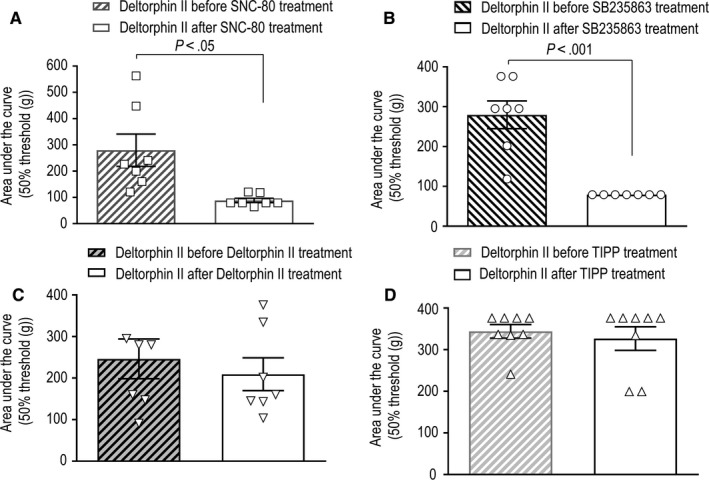
Repeated administration of DOPr agonists induces different degrees of heterologous tolerance. Rats suffering from diabetic neuropathy were injected i.t. with deltorphin II (EC_50_: 5.22 M) one day prior and one day after six consecutive injections of (A) SNC‐80 (0.55 nmol/30 μL), (B) SB235863 (1.7 nmol/30 μL), (C) deltorphin II (0.30 nmol/30 μL) or (D) TIPP (0.40 nmol/30 μL). Pressure withdrawal thresholds were measured immediately after administration of the probe and then every 15 min until return to baseline. Results are expressed as mean area under the curve ± SEM and correspond to 7 rats/group. Statistical comparisons of the response to deltorphin II before the first and after the last day of treatment were performed using two‐tailed Student's *t* test to reveal a difference in rats treated for six days with SNC‐80 (*P* = .0124) and SB235863 (*P* = .0012), but not with deltorphin II (*P* = .4202) or TIPP (*P* = .6102)

Ligand signalling efficacy affects the development of chronic tolerance simply because full agonists require lower occupancy than partial ones to induce analgesia,^38^, ^39^ allowing for ‘spare receptors’. Hence, we sought to corroborate development of chronic tolerance without introducing efficacy as confounding factor. For this purpose, we assessed how repeated administration of ED_80_ doses of each of the four agonists influenced the analgesic response to a common probe. Deltorphin II, which was used as the common test ligand, was injected at a submaximal dose (ED_50_) one day prior to the beginning of each treatment and a day after the last injection of each agonist (see Figure 1 for experimental design). By comparing the effect of this fixed dose of deltorphin II before and after each treatment, it was possible to corroborate that SNC‐80 (Figure 5A) and SB235863 (Figure 5B) induced cross‐tolerance to the common probe. On the other hand, chronic treatment with deltorphin II (Figure 5C) and TIPP (Figure 5D) was without effect, confirming that internalization profiles are not predictive of chronic tolerance to a heterologous agonist. Mechanical thresholds were established daily after injection of different agonists. Although behavioural tolerance has been described with this administration schedule,^40^ this type of tolerance would be expected to appear for all treatments. It is therefore unlikely that tolerance that only appears for internalizing agonists could be explained by habituation.

**Figure 6:**
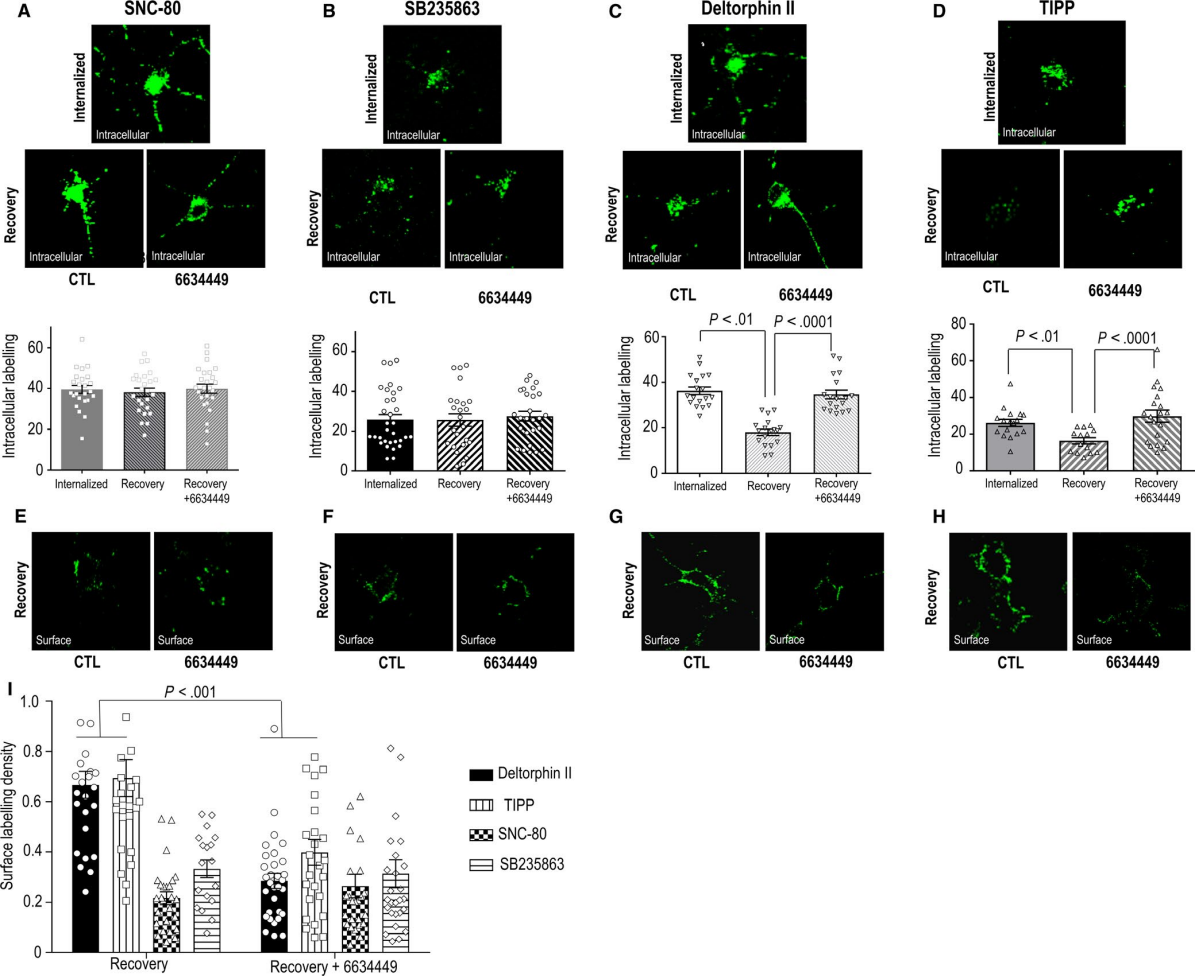
DOPr agonists have different recycling profiles that are distinctively influenced by ECE2 activity. Primary cortical neuron cultures were transfected with Flag‐DOPrs and incubated with primary antibody prior to exposure to either vehicle or indicated agonists (10 µmol/L, 60 min). A‐D, At the end of treatment, cells were either immediately processed for intracellular labelling (panels below) or allowed to recover from treatment (60 min; lower panels) in the presence or absence of ECE2 blocker (6634449; 20 μmol/L) as indicated. Histograms below the images show intracellular labelling density ± SEM (arbitrary units) for the total number of neurons quantified per condition immediately after treatment or following recovery in the presence or absence of 6634449 as indicated. Data were generated in three independent experiments. Mean intracellular labelling density following internalization, recovery and recovery in presence of 6634449 were compared for each agonist using one‐way ANOVA. Post hoc comparisons using Sidak's test revealed no effect of recovery or of the ECE2 inhibitor for SNC‐80 and SB23586. Deltorphin II and TIPP showed both an effect of recovery and of the ECE2 inhibitor, as indicated. E‐H, Another set of neurons was treated as above and stripped of all surface labelling before allowing them to recover in the presence or absence of 6634449 as indicated. At the end of recovery, cells were processed for surface labelling. I, Histograms show surface labelling density ± SEM (arbitrary units) in cells that were allowed to recover in the presence or absence of 6634449 following treatment with different agonists. Data were generated in three independent experiments, and the total number of neurons quantified per condition is shown in the figures. Data were analysed with two‐way ANOVA to compare membrane labelling following recovery from exposure to different agonists in the presence or absence of ECE2 inhibitor. Analysis showed an effect of agonist (*P* < .001), an effect of ECE2 inhibitor (*P* < .001) and an interaction between both factors (*P* ˂ .001). Post hoc comparisons using Sidak's test revealed an effect of ECE2 inhibitor for deltorphin II and TIPP, *P* = .001

We had previously shown that recycling is essential for maintaining analgesic responses following two consecutive acute injections of DOPr agonists.^19^ Hence, we wanted to determine if preservation of analgesic response over repeated treatment was also associated with ligand ability to support recycling. To address this question, we first used a previously validated approach to compare DOPr recycling by different agonists.^19^ In particular, primary cortical neuron cultures were transfected with Flag‐DOPrs, and on the day of the experiment, receptors were labelled at the cell surface. Cultures were then exposed to different agonists (10 µmol/L, 60 minutes) to induce internalization. At the end of treatment, neurons were washed to remove ligand and antibody bound to non‐internalized receptors. After wash, neurons were either immediately processed for quantification of intracellular Flag‐DOPrs or were allowed to recover 60 minutes in the absence of ligand before revealing intracellular immunoreactivity. For SNC‐80 (Figure 6A) and SB235863 (Figure 6B), the amount of intracellular immunoreactivity present at the end of the recovery period was not different from the one observed immediately after the end of treatment, indicating that internalized receptors remained trapped within the intracellular compartment even one hour after removal of these agonists from the incubation medium. On the other hand, in cultures exposed to deltorphin II (Figure 6C) or TIPP (Figure 6D) mean intracellular labelling intensity was significantly reduced following recovery in the absence of ligand, indicating that receptors internalized by these agonists left the intracellular compartment upon removal of the drug. To corroborate that receptors leaving the cytoplasm were relocated to the membrane, immunoreactivity following recovery was also assessed in non‐permeabilized neurons (Figure 6E‐H). Consistent with intracellular labelling patterns, surface labelling after recovery was less for SNC‐80 and SB235863 than for deltorphin II and TIPP (Figure 6I). Thus, taken together, these results show that persistence of analgesic responses upon repeated drug administration persists for recycling ligands independent of the extent of their internalization capacity.

### 3.3 Recycling is essential for persistence of analgesic response over repeated exposure to DOPr agonists

A major distinction between agonists that induced tolerance over repeated administration versus those that did not is their chemical structure. Indeed, deltorphin II is a naturally occurring opioid peptide^32^ and TIPP is an opioid peptide analogue.^33^ On the other hand, SNC‐80^34^ and SB235863^35^ are synthetic, non‐peptide ligands. Several of the naturally occurring opioid peptides are internalized with the receptor and can be hydrolysed by the endothelin‐converting enzyme 2 (ECE2), an endosomal protease that functions at acidic pH.^36^ Deltorphin II is one of the ECE2 substrates, and inhibition of the enzyme interferes with recycling of deltorphin II‐activated DOPrs.^35^ We took advantage of this knowledge to determine whether interfering with recycling had any impact on the development of chronic analgesic tolerance by the different agonists. In a first series of experiments, we assessed whether the ECE2 inhibitor 6634449 had any effect on recycling by the different agonists. We observed that 6634449 was without effect on the redistribution of internalized receptors during recovery from SNC‐80 (Figure 6A,E) or SB235863 (Figure 6B,F) treatments, but caused those internalized by deltorphin II (Figure 6C,G) and TIPP (Figure 6D,H) to remain trapped in the cytosol, blocking their recovery at the cell surface (Figures6G ‐6H).

**Figure 7:**
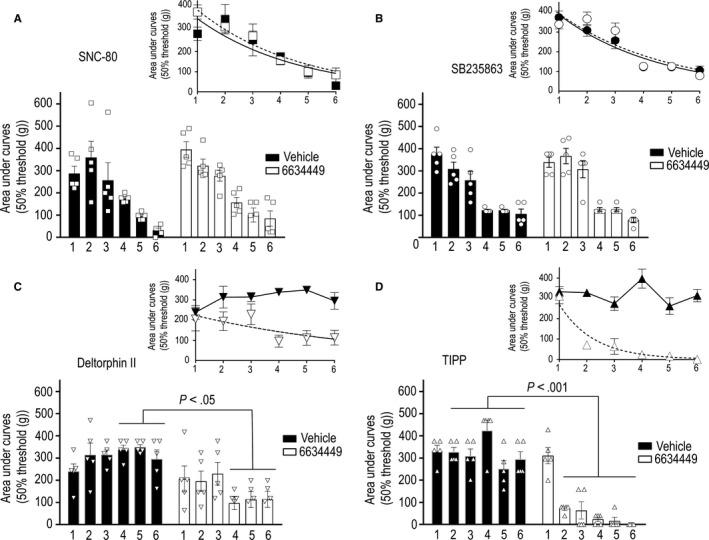
Inhibition of ECE2 activity precipitates tolerance for ligands that support recycling. Twenty min before daily i.t. administration of (A) SNC‐80 (0.55 nmol/30 μL), (B) SB235863 (1.7 nmol/30 μL), (C) deltorphin II (0.30 nmol/30 μL) or (D) TIPP (0.40 nmol/30 μL), rats suffering from diabetic neuropathy received i.t. injections of either ECE2 inhibitor 6634449 (3 nmol/30 μL) or vehicle (CSF). Pressure withdrawal thresholds for mechanical allodynia were measured at 15‐min intervals immediately after each administration until return to baseline, and the area under the curve was consigned each day. Results are expressed as mean area ± SEM and correspond to five rats/group. Statistical comparisons using two‐way ANOVA revealed an effect of time for SNC‐80 (*P* ˂ .0001) and SB235863 (*P* ˂ .0001) but no effect of the ECE2 inhibitor nor interaction. Comparisons for deltorphin II revealed no effect of time, an effect of the ECE2 inhibitor (*P* ˂ .0001) and no interaction. Post hoc Sidak's comparisons revealed differences indicated in the figure. Comparisons for TIPP revealed an effect of time (*P* ˂ .0001), an effect of the ECE2 inhibitor (*P* ˂ .0001) and their interaction (*P* ˂ .0001). Results of post hoc Sidak's comparisons are shown in the figure. Insets show the kinetics of analgesic response in the presence (dashed line) and absence (full line) of 6634449

**Figure 8:**
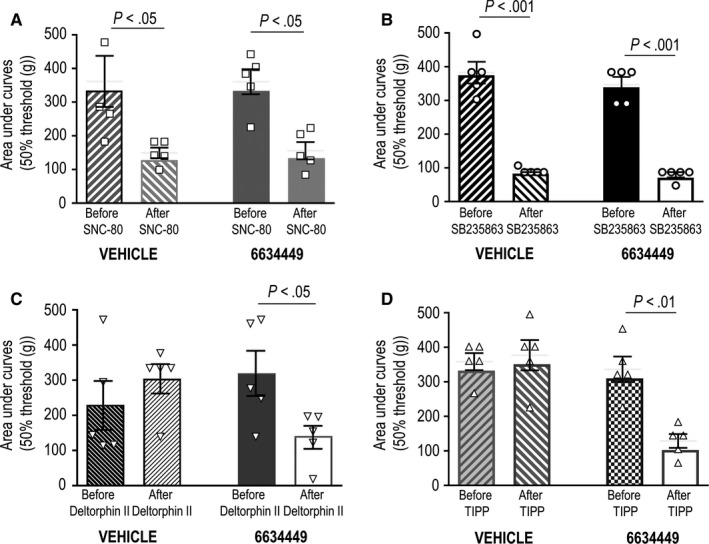
Receptor recycling protects from heterologous tolerance. Rats suffering from diabetic neuropathy were injected i.t. with deltorphin II (EC50: 5.22 mol/L) the day prior and the day after a six‐day treatment with (A) SNC‐80 (0.55 nmol/30 μL), (B) SB235863 (1.7 nmol/30 μL), (C) deltorphin II (0.30 nmol/30 μL) or (D) TIPP (0.40 nmol/30 μL). Twenty min prior to each of the six injections of indicated agonists, rats were administered ECE2 inhibitor 6634449 or vehicle (CSF) as indicated. Results are expressed as mean area under the curve ± SEM and correspond to five rats/group. Statistical comparisons using two‐way ANOVA revealed an effect of SNC‐80 (*P* ˂ .01) and SB235863 (*P* ˂ .0001) treatments but no effect of the ECE2 inhibitor nor interaction. Deltorphin II‐treated animals showed an interaction (*P* ˂ .0001), and post hoc Student's *t *test revealed an effect of 6634449 as indicated in the figure. Comparisons for TIPP revealed an effect of the agonist (*P* ˂ .05), an effect of the ECE2 inhibitor (*P* ˂ .01) and an interaction (*P* ˂ .05). Post hoc Student's *t* test revealed an effect of 6634449 as indicated in the figure

Having established that the ECE2 inhibitor blocked recycling supported by deltorphin II and TIPP, we reasoned that if chronic analgesic tolerance was prevented by recycling, then the administration of 6634449 together with these agonists should precipitate tolerance. Conversely, as the ECE2 inhibitor did not change intracellular accumulation of receptors internalized by SNC‐80 or SB235863, their co‐administration with the ECE2 inhibitor would not be expected to influence the time course of their analgesic response. In effect, when 6634449 (0.1 mmol/L; i.t.) was administered 20 minutes prior to each injection of synthetic agonists, tolerance induced by SNC‐80 or SB235863 was similar to that observed in controls pre‐injected with CSF (Figure 7A,B), and curves representing decay of analgesic response in presence or absence of the ECE2 inhibitor were superimposed (Figure 7A,B, insets). In particular, analgesic *t*_1/2_ for SNC‐80‐treated rats receiving vehicle was within a 95% CI of 1.3 to 4.9 days, and for those pre‐treated with the ECE2 inhibitor, the *t*_1/2_ of analgesic response was 1.4 to 3.1 days (*P* = .6294; time constants compared using global curve fitting with shared parameters). Similarly, analgesic *t*_1/2_ for SB235863 in 6634449‐treated rats (95% CI = 1.8‐3.8 days) did not differ from that of animals pre‐injected with vehicle (95% CI = 1.2‐3.3 days; *P* = .6586). On the other hand, analgesia by deltorphin II and TIPP was significantly shorter in animals receiving the ECE2 inhibitor (Figure 7C,D). Thus, whereas analgesic responses by either agonist displayed no measurable decay *t*_1/2_ in animals pre‐treated with vehicle, analgesic effects of TIPP and deltorphin II were, respectively, reduced by day 2 (*P* = .001) and day 4 (*P* < .02) in animals previously exposed to 6634449 (Figure 7C,D). In 6634449‐treated animals, analgesic *t*_1/2_ for the partial agonist TIPP (95% CI = 0.6‐1.4 days) was shorter than that of SNC‐80 CI = 1.4‐3.1 days; *P* ˂ .0001 or SB235863 (CI = 1.8‐3.8 days; *P* < .0001), while that of deltorphin II remained ill‐defined despite the observed loss of its analgesic effects (Figure 7). ECE2 activity was also essential for preventing TIPP from inducing heterologous tolerance. Indeed, whereas heterologous tolerance by SNC‐80 (Figure 8A) and SB235863 (Figure 8B) remained unchanged by 6634449 administration, in rats that received TIPP plus the ECE2 inhibitor, the analgesic response produced by deltorphin II (ED_50_) was significantly reduced as compared to rats that received TIPP plus vehicle (Figure 8D). As expected, co‐administration of 6634449 with deltorphin II over a six‐day period reduced analgesic effect of the drug's ED_50_ (Figure 8C).

## 4 DISCUSSION

In the present study, we used a model of diabetic neuropathy to determine whether ligand‐specific trafficking profiles were predictive of DOPr agonist potential to induce analgesic tolerance. We found that ligands that supported receptor recycling to the membrane had sustained anti‐allodynic effect over a 6‐day administration schedule, and we further established that recycling was necessary and sufficient to prevent the loss of analgesic responses over repeated administration.

Because of their constitutive interaction with GASP‐1, a sorting protein that excludes receptors from the recycling path and directs them to lysosomes,^41^, ^42^ DOPrs have been classically considered as committed for degradation.^43^ If direct sorting to lysosomes was the only itinerary followed by these receptors, then internalizing ligands would systematically promote degradation of the receptor and induce analgesic tolerance. The highly internalizing agonist SNC‐80, whose acute^16^, ^18^ and repeated administration^18^ induces marked analgesic tolerance, typically represents this type of ligand. At the same time, other DOPr agonists that display similar internalization capacity as SNC‐80^37^ fail to induce acute tolerance.^16^, ^17^, ^19^ Recent studies have shown that these agonists support recycling by various mechanisms. In particular, the enkephalin analogue DPDPE and the naturally occurring ligand deltorphin II which fail to induce acute analgesic tolerance, respectively, promote DOPr recycling through transient interaction with βarr2^16^, ^19^ or via ligand degradation by ECE2.^35^ Here, we show that agonists that support DOPr recycling also maintain analgesic response over repeated administration. Moreover, for the two peptidic agonists tested (TIPP and deltorphin II), ECE2 activity was essential not only for membrane recovery of internalized receptors but also for protection from chronic tolerance, causally associating both events.

Sequestration profiles had no predictive value with respect to the decay of analgesia over repeated administration but, on the other hand, internalization capacity was inversely associated with the duration of a single analgesic dose of DOPr agonists. Indeed, the time course of acute analgesia induced by the injection of poorly internalizing ligands with low efficacy/potency profiles like TIPP and SB235863 was longer than analgesia induced by highly internalizing, efficacious agonists like SNC‐80 and deltorphin II. These observations are not only consistent with previous observations showing that decay of signalization is quicker for DOPr ligands that promote maximal sequestration,^36^ but also with the notion that DOPrs must remain at the membrane to engage Kir3 and Cav2 channels effectors which mediate analgesia.^1^ Interestingly, signalling efficacy or potency had no obvious association with time course of chronic tolerance. Indeed, chronic tolerance did not develop for full agonist deltorphin II nor for partially effective TIPP, although it rapidly appeared following repeated administration of the full agonist SNC‐80 and low potency agonist SB235863. Interestingly, upon inhibition of recycling, analgesia by the least efficacious agonist TIPP decayed with the shortest t_1/2_ among all agonists tested, underlining the important contribution of recycling in maintaining prolonged analgesia by this partial, affinity‐driven agonist.^1^

Agonists that do not produce tolerance over repeated administration are highly desirable for chronic pain management. However, DOPr agonists that rely on recycling for sustained analgesic actions are all peptide ligands,^16^, ^17^, ^19^, ^35^ and poor biodisponibility and restricted brain penetration represent a clear obstacle for clinical application. Non‐peptide DOPr agonists like JNJ‐20788560,^12^ morphine‐6‐O‐sulphate (M6S)^5^ and PN6047^44^ induce sustained analgesic response for a similar time period as the recycling peptide agonists assessed in this study, but the mechanism underlying this prolonged analgesia remains to be elucidated. JNJ‐20788560 and PN6047 efficacy to induce G protein activation is comparable to that of the full agonist SNC‐80. On the other hand, their internalization capacity is markedly less than that of this standard ligand.^44^, ^45^, ^46^ As DOPr‐βarr interaction is a major determinant of DOPr internalization^1^ and given that DOPr recycling requires unstable DOPr‐βarr association, a plausible mechanism for these non‐peptidic ligands to support recycling is weak interaction between the two proteins which interferes with maximal internalization but simultaneously promotes recycling.

In summary, the study provides evidence that DOPr agonists that support receptor recycling to the membrane can produce sustained analgesic responses over repeated administrations.

## CONFLICT OF INTEREST

None.

## AUTHORS' CONTRIBUTIONS

HBT and YH have tested mechanical allodynia in rats and HBT interpreted the results. HBT and IC performed the primary neuronal cultures. IC did the quantification of DOPr trafficking in primary neuron cultures. GP was responsible for the supervision of project and wrote the manuscript with HBT and RC.

## Data Availability

The data that support the findings of this study are available from the corresponding author upon reasonable request.
